# Treatment of pulmonary arterial hypertension in patients with connective tissue diseases: a systematic review and meta-analysis

**DOI:** 10.1007/s11739-024-03539-1

**Published:** 2024-02-20

**Authors:** Mustafa Erdogan, Sinem Nihal Esatoglu, Burcak Kilickiran Avci, Gulen Hatemi

**Affiliations:** 1 Division of Rheumatology, Department of Internal Medicine, Cerrahpaşa Medical School, Istanbul University-Cerrahpaşa, Istanbul, Turkey; 2 Department of Cardiology, Cerrahpaşa Medical School, Istanbul University-Cerrahpaşa, Cerrahpaşa Campus, Kocamustafapaşa Cad. No: 34/E, Fatih, 34998 Istanbul, Turkey

**Keywords:** Pulmonary arterial hypertension, Systemic lupus erythematosus, Systemic sclerosis, Connective tissue disease, Meta-analysis, Vasodilator treatment

## Abstract

The evidence for the treatment of connective tissue disease-associated pulmonary arterial hypertension (CTD-PAH) mostly depends on subgroup or post hoc analysis of randomized controlled trials (RCTs). Thus, we performed a meta-analysis of RCTs that reported outcomes for CTD-PAH. PubMed and EMBASE were searched for CTD-PAH treatment. The selected outcomes were functional class (FC) change, survival rates, 6-min walk distance (6-MWD), clinical worsening (CW), N-terminal prohormone BNP (NT-proBNP), pulmonary vascular resistance (PVR), mean pulmonary arterial pressure (mPAP), right atrial pressure (RAP), and cardiac index (CI). The meta-analysis was conducted according to the PRISMA guidelines and registered in PROSPERO (CRD42020153560). Twelve RCTs conducted with 1837 patients were included. The diagnoses were systemic sclerosis in 59%, SLE in 20%, and other CTDs in 21%. The pharmacological interventions were epoprostenol, treprostinil, sildenafil, tadalafil, bosentan, macitentan, ambrisentan, riociguat, and selexipag. There was a significant difference between interventions and placebo in FC, 6MWD, CW, PVR, RAP, and CI that favored intervention. Our analysis showed a 39% reduction in the CW risk with PAH treatment. The short-term survival rates and mean serum NT-proBNP changes were similar between the study and control groups. Treatment for CTD-PAH had favorable effects on clinical and hemodynamic outcomes but not on survival and NT-proBNP levels. Different from the previous meta-analyses that focused on 6-MWD, time to clinical worsening, and CW as outcomes, this meta-analysis additionally reports the pooled analysis of change in FC, hemodynamic measurements (RAP, PVR, CI), and NT-proBNP, some of which have prognostic value for PAH.

**Supplementary Information:**

The online version contains supplementary material available at 10.1007/s11739-024-03539-1.

## Introduction

Pulmonary hypertension (PH) is defined as increased mean pulmonary arterial pressure (mPAP) > 20-mmHg by right heart catheterization (RHC) during rest and is classified into 5 groups based on etiology [1]. Group 1 PH, also called pulmonary arterial hypertension (PAH), is the pre-capillary form and the most common form of PH. Other PH groups are group 2 PH (due to left heart disease), group 3 (due to lung diseases and/or hypoxia), group 4 (chronic thromboembolic PH and PH due to other pulmonary artery obstructions), and group 5 (PH with unclear and/or multifactorial mechanisms) [1].

Idiopathic PAH (IPAH) is the most common type of PAH in non-endemic schistosomiasis areas such as Europe and North America, with an estimated prevalence of 5–52 per million [1, 2]. Among the etiologies of associated PAH, the most common etiology is connective tissue diseases (CTDs). The frequency and prognosis of PAH is different across CTDs. Systemic sclerosis (SSc) is the leading cause of CTD-PAH, and 8–12% of SSc patients develop PAH [2, 3]. The main pathogenetic mechanisms in SSc-PAH are thought to be endothelial dysfunction and impaired balance between vasoconstrictor and vasodilator mediators such as endothelin-1 (ET-1), nitric oxide (NO), prostacyclin (PG-I2), and smooth muscle proliferation leading to vasculopathy [4, 5]. The exact prevalence of PAH among patients with other types of CTDs, such as systemic lupus erythematosus (SLE), mixed connective tissue disease (MCTD), and Sjögren’s Syndrome, is not clear due to the lack of routine screening in these patients. However, the reported estimated prevalence is much less than that of SSc (< 1%) [3, 6, 7].

The mortality risk of patients with PAH-SSc is four times higher than that of patients with IPAH. The 1- and 3-year survival rates were 88% and 49% among patients with PAH-SSc and 95% and 84% among patients with IPAH [8]. Factors affecting the higher mortality rate in PAH-SSc patients may be concomitant interstitial lung disease (ILD), left heart dysfunction due to myocardial involvement, increased incidence of pulmonary veno-occlusive disease, and increased incidence of atherosclerosis in patients with SSc [9–12]. This higher risk raised the question of whether PAH-specific treatment modalities are equally effective in patients with CTD-PAH and IPAH. On the other hand, as a result of severe micro vasculopathy, digital ulceration (DU), which is not fatal but a symptom associated with impaired quality of life in SSc, occurs in half of the patients during the course of the disease. Considering that the development of digital ulcers and PAH in SSc share similarities in pathogenesis, treatment options are also similar for both manifestations of SSc [13, 14].

The options for managing PAH have expanded in the last two decades. The available options are endothelin receptor antagonists (ERA) (ambrisentan, bosentan, and macitentan), phosphodiesterase type 5 (PDE-5) inhibitors (sildenafil and tadalafil), soluble guanylate cyclase stimulators (riociguat), prostacyclin analogs (epoprostenol, treprostinil, iloprost) and a prostacyclin receptor agonist (selexipag) [15–19].

However, there are conflicting results for the treatment responses of patients with CTD-PAH and IPAH in randomized controlled trials (RCTs) [10, 19–22]. Most RCTs were not specifically designed for CTD-PAH patients, and these patients were generally represented as a subgroup. Furthermore, these studies were not powered to analyze CTD-PAH patients separately, and in addition to the insufficient number of patients with CTD-PAH patients included in PAH studies, baseline characteristics were not stratified according to the underlying etiology. Another issue is the evolution of the outcomes used in PAH studies. Outcomes associated with mortality and morbidities have been more commonly assessed in recent years. To overcome these limitations, a number of meta-analyses with different methodologies and time periods have been conducted to evaluate the magnitude of the benefit of PAH therapies in CTD-PAH patients [19, 20, 23]. The reported meta-analyses mainly focused on the six-minute walk distance (6-MWD), time to clinical worsening (TTCW), and the risk of clinical worsening (CW) as outcomes. However, other important outcomes such as changes in FC, N-terminal prohormone BNP (NT-proBNP) levels, and cardiopulmonary hemodynamic measurements, including pulmonary vascular resistance (PVR), right atrial pressure (RAP), and cardiac index (CI), were not covered. These outcomes comprise the main parameters in the follow-up of patients with PAH.

We conducted a systematic literature review and meta-analysis of RCTs to evaluate the clinical and hemodynamic efficacy of PAH-specific therapies for CTD-PAH patients.

## Material and methods

### Protocol

We conducted this systematic review and meta-analysis according to the PRISMA guidelines. The protocol was registered in the International Prospective Register of Systematic Reviews (PROSPERO) with the registration number CRD42020153560.

### Literature search strategy

PubMed and EMBASE were searched using the keyword combinations “pulmonary hypertension OR pulmonary arterial hypertension OR PAH” from its inception until June 2022. After a restriction for RCTs, two authors (ME and SNE) independently screened the titles and abstracts using the COVIDENCE platform. The full texts of articles that potentially met eligibility criteria at the first screening stage were evaluated for inclusion criteria. In case of disagreements, the senior author (GH) made the final decision.

### Inclusion and exclusion criteria

Inclusion criteria were as follows: (1) study design: RCTs, (2) patients: Participants with a diagnosis of CTD-PAH, (3) interventions: Studies that assessed PAH-specific treatment modalities comprising ambrisentan, bosentan, macitentan, epoprostenol, treprostinil, iloprost, sildenafil, tadalafil, riociguat, selexipag as monotherapy or as dual or triple combinations; (4) controls: Studies that included placebo, or PAH-specific agents used as monotherapy, or combination therapy as controls; (5) outcomes: change from baseline in World Health Organization FC (WHO-FC), 6-MWD, NT-proBNP, PVR, mean pulmonary arterial pressure (mPAP), RAP, and CI; survival and CW rates. Different definitions were used for CW across studies (Online Table S3). During the analyses, we accepted the definition of CW as indicated in each trial. We excluded RCTs that did not report the results for CTD-PAH patients and open-label extension phases of the studies.

### Data extraction

To reduce bias in reporting and error in data collection, two researchers extracted data from the included studies independently. The items entered into an Excel file were presented in Table S1 (Online Resource). The most inclusive or recent publication was selected when there was more than one publication based on the same study.

### Quality assessment

Quality assessment of the articles was done using the revised Cochrane risk-of-bias tool for randomized trials (RoB 2) [24].

### Statistical analyses

The RevMan 5.4.1 software was used for meta-analyses as suggested by Cochrane Collaboration. Odds ratios (OR) and mean differences (MD) with 95% CI were used to estimate the effect size. The heterogeneity of the trials was tested by the I^2^ test. The random effects model was used to test observed treatment effect estimates due to the heterogeneity of the characteristics of the included patients, such as background therapy, WHO-FC, the proportion of CTD subgroups, and the definition of clinical worsening.

The missing data for mean differences or standard deviations were imputed by missing data replacing methods [25, 26].

## Results

### Results of the search

**Fig. 1:**
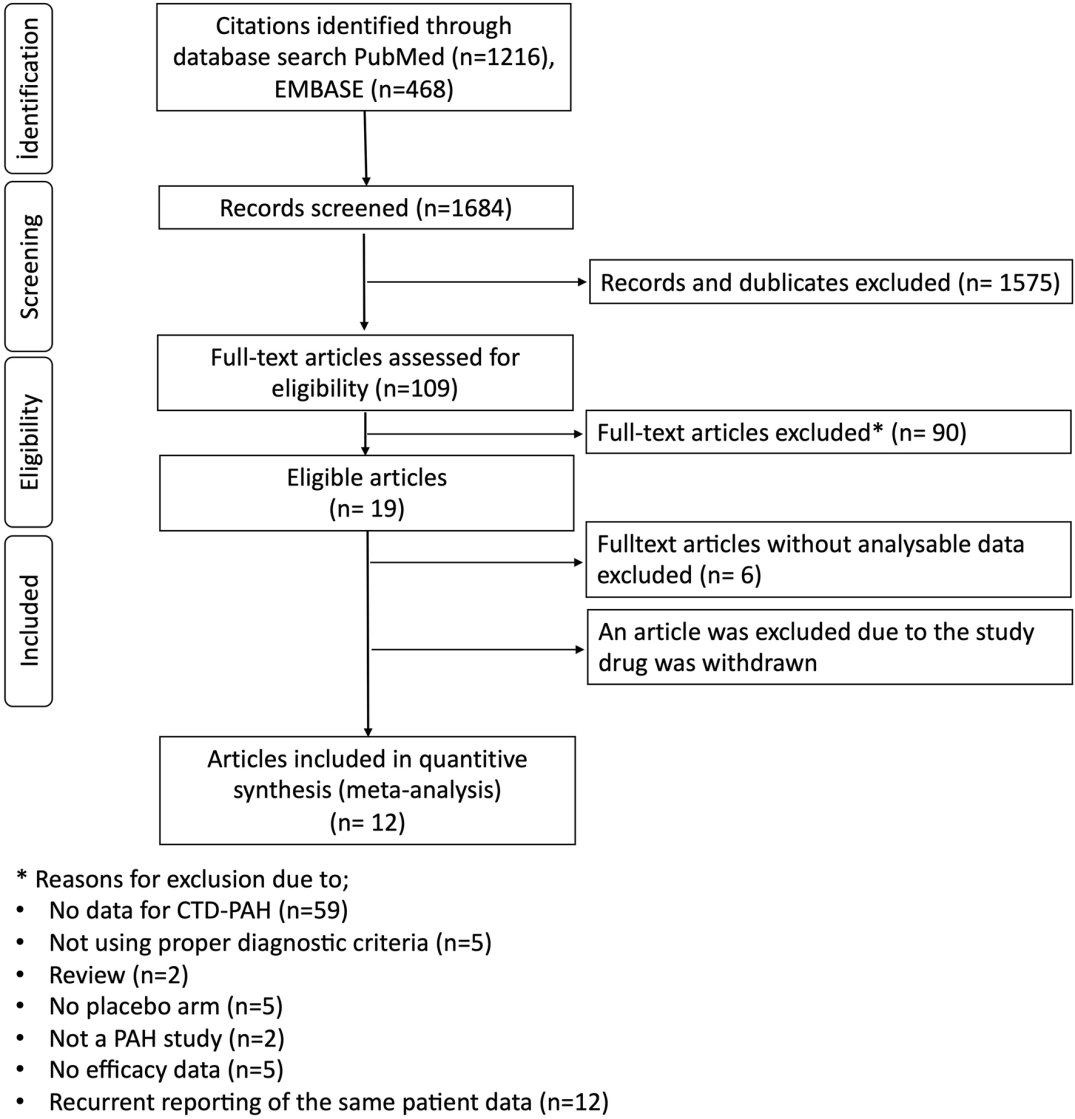
Flow chart of the study inclusion process

Initial PubMed and EMBASE searches revealed 1216 and 468 articles, respectively. After reading the titles and abstracts, 1107 articles were excluded, and the full texts of the remaining 109 articles were reviewed in detail. Among these 109 articles, 19 RCTs reported outcome data for CTD-PAH. One study with a drug (sitaxentan) that was withdrawn from the market worldwide due to fatal hepatotoxicity was excluded [27]. After excluding 6 RCTs due to unavailable data for the meta-analyses, the remaining 12 RCTs were included in the meta-analyses (Fig. 1).

### Study and patients’ characteristics

Among the 18 studies that had recruited 2230 patients with CTD-PAH, 2 studies had been conducted among only CTD-PAH patients [28, 29], 9 studies included patients with CTD-PAH in addition to those with IPAH and provided results of the CTD-PAH patients separately [30–38], and 7 studies were post hoc analyses of PAH studies providing outcomes of CTD-PAH subgroups [33, 39–45]. Table 1 and Table S1 (Online Resource) show the 12 RCTs characteristics included in the meta-analyses, and Table S2 (Online Resource) shows the characteristics of the RCTs published over a 21-year period. The patients had a mean age of 50.3 years. Most patients were female (82%) and in NYHA FC III (62%). All of these RCTs included PAH patients with mPAP > 25 mmHg and PCWP ≤ 15 mmHg with a variable PVR cut-off (no PVR criteria to ≥ 6WU). (Table S2-Online Resource).

**Table 1:** Study characteristics of the RCTs included in the meta-analysis

First author, year	Official acronym	Trial	Control	Trial	Control
Badesch DB, 2000	–	Epoprostenol iv + conventional therapy	Conventional therapy alone	55	55
Oudiz RJ, 2004	–	Treprostinil sc	Placebo	41	49
Denton CP, 2006	BREATHE-1	Bosentan	Placebo	44	22
Badesch DB, 2007	SUPER-1	Sildenafil 20, 40, or 80 mg tid	Placebo	62	22
Galie S, 2008	AIRES 1–2	Ambrisentan	Placebo	81	43
Barst RJ, 2011	PHIRST-1	Tadalafil 20 mg or 40 mg	Placebo	36	16
Channick RN, 2014	SERAPHIN	Macitentan 3 mg or 10 mg	Placebo	143	164
McLaughlin V, 2015	COMPASS-2	Bosentan 62.5–125 mg bid	Placebo	43	35
Coghlan JG, 2017	AMBITION	Amb. 10 mg + Tad. 40 mg	Amb. 10 mg or Tad. 40 mg	103	84
Humbert M, 2017	PATENT 1- 2	Riociguat up to 1.5 or 2.5 mg tid	Placebo	151	66
Gaine S, 2017	GRIPHON	Selexipag 200–1600 μg bid*	Placebo	167	167
White RJ, 2020	FREEDOM-EV	Treprostinil 0.125 mg tid	Placebo	94	84

First author, year	Primary outcome	Trial duration (weeks or months)	Etiology, n (%)		
			SSc	SLE	Others
Badesch DB, 2000	Δ6-MWD (meters)	12 wk	47 (80)	16 (20)	0
Oudiz RJ, 2004	Δ6-MWD (meters)	12 wk	19 (45)	16 (38)	7 (17)
Denton CP, 2006	Δ6-MWD (meters)	16 wk	52 (79)	8 (12)	6 (9)
Badesch DB, 2007	Δ6-MWD (meters)	12 wk	NR	NR	NR
Galie S, 2008	Δ6-MWD (meters)	12 wk	NR	NR	NR
Barst RJ, 2011	Δ6-MWD (meters)	16 wk	NR	NR	NR
Channick RN, 2014	TTFH	36 mo	NR	NR	NR
McLaughlin V, 2015	TTFM	16 wk	NR	NR	NR
Coghlan JG, 2017	TTCW	12 wk	118 (63)	17 (9)	52 (28)
Humbert M, 2017	6MWD (LS mean difference)	12 wk	NR	NR	NR
Gaine S, 2017	TTCW	26 wk	170 (51)	82 (24.5)	82 (24.5)
White RJ, 2020	TTCW	24 wk	NR	NR	NR

*200–400 μg: 16.6%, 600–1000 μg: 27%, 1200–1600 μg: 44.9%

*Amb* Ambrisentan, *Tad* Tadalafil, *sc* subcutaneous, *iv* intravenous, *mg* milligram, *RCT* randomized clinical trials, *Δ 6-MWD* change in 6-min walk distance, *LS* least square, *mg* milligram, *NR* Not reported, *TTCW* time to clinical worsening, *TTFH* time to first hospitalization, *TTFM* time to first mortality/morbidity event, *wk* weeks, *SLE* systemic lupus erythematosus, *SSc* systemic sclerosis

#### Efficacy outcomes

The meta-analyses evaluated the effect of mono or combination PAH therapies on FC, 6-MWD, NT-proBNP level, PVR, RAP, and CI measured between 12 and 24 weeks and the risk of clinical worsening.

##### Functional class

**Fig. 2:**
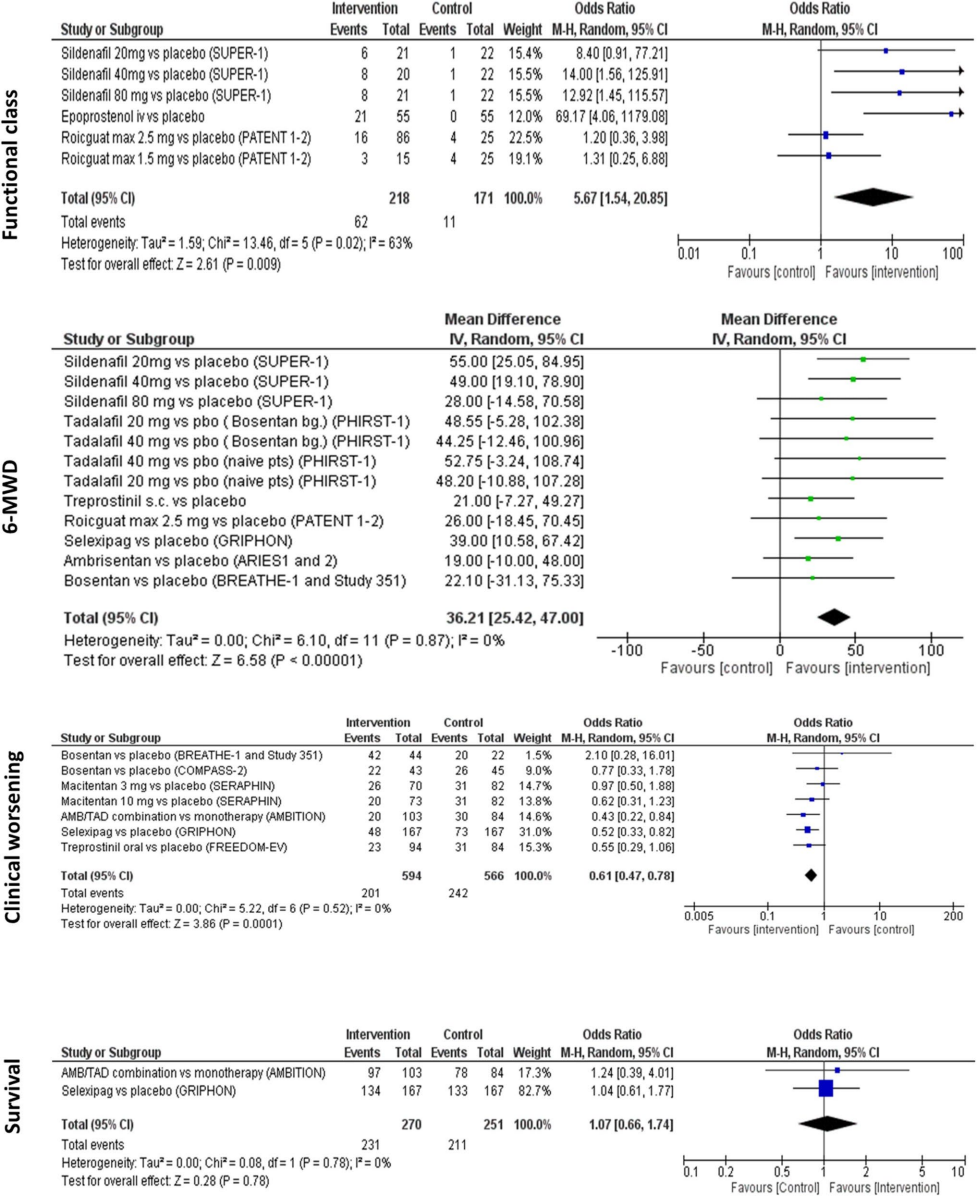
The results of the meta-analyses of the clinical outcomes

The proportion of patients with improved FC (WHO or NHYA-FC) was an outcome in 3 RCTs [28, 40, 44]. There were 6 treatment dose subgroups with 389 patients. The heterogeneity between studies was moderate (I^2^ 63%, p = 0.02). The pooled analysis of these patients revealed that the probability of having an improved FC was significantly higher in intervention groups (28.4% vs. 6.4%, OR 5.67, 95% CI 1.5–20.8, Z = 2.61, p = 0.009) (Fig. 2).

##### 6-MWD

6-MWD was the most frequently used outcome reported in 12 RCTs [17, 18, 21, 26–28, 32, 33]. However, only 7 studies, including 12 subgroups and 921 patients, provided sufficient data for meta-analysis [18, 21, 26–28, 32, 33]. The heterogeneity between these trials was low (I^2^ = 0%, p = 0.87). The placebo or monotherapy corrected mean difference was 36.2 m (95% CI 25–47, Z = 6.58, p < 0.001), favoring the intervention group (Fig. 2).

##### Clinical worsening

CW was an outcome as a combined endpoint consisting of multiple parameters in 6 RCTs [32, 33, 39, 41, 42, 45]. The definitions of the CW in these trials were summarized in Table S3 (Online Resource). There was a low heterogeneity between trials (I^2^ = 0%, p = 0.52). The pooled analysis of the 7 subgroups in these trials revealed that 34% (n = 201/594) of the patients in the intervention group and 43% (n = 242/566) of the patients in the control group had CW. The risk reduction was 39% (OR 0.61, 95% CI 0.47–0.78, Z = 3.9, p < 0.001). Combination therapies (COMPASS-2, AMBITION, and FREEDOM-EV) provided an even more pronounced risk reduction (46% risk reduction, OR 0.54, 95% CI 0.36–0.82, Z = 2.94, p = 0.003; I^2^ = 0%, p = 0.58).

##### Survival

Survival was reported as an outcome in 2 RCTs with a follow-up time of 24 and 26 weeks [33, 42]. There was a low heterogeneity between trials (I^2^ = 0%, p = 0.78). The pooled analysis of these trials revealed a similar survival rate in the intervention and control groups (OR 1.07, 95% CI 0.66–1.74, Z = 0.28 p = 0.78] (Fig. 2).

##### NT-proBNP

**Fig. 3:**
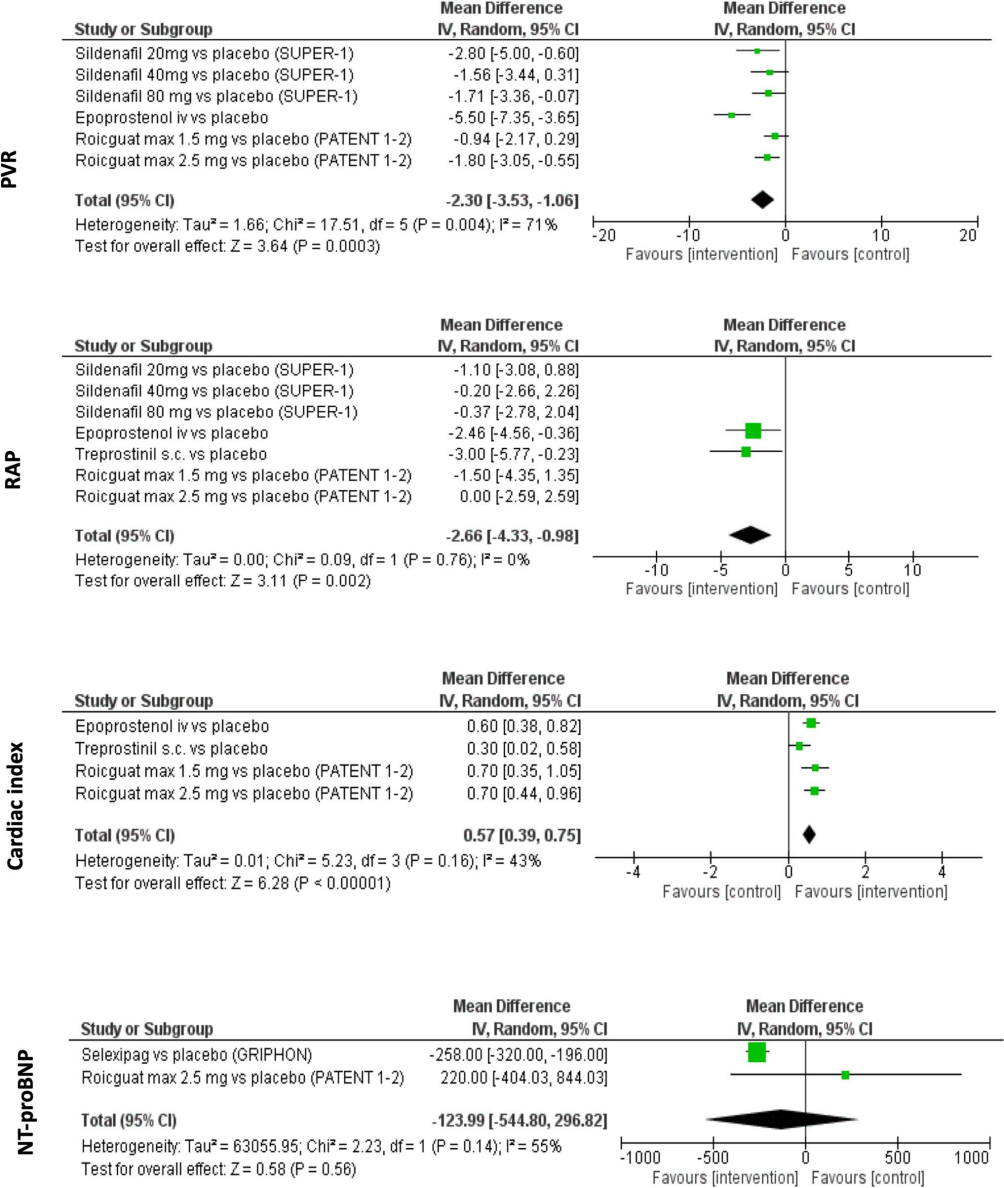
The results of the meta-analyses of the hemodynamic outcomes and NT-proBNP

Three RCTs reported a change in serum NT-proBNP level as an outcome [33, 42, 44]. In the meta-analysis of the 2 RCTs with available data evaluating the effect of selexipag and riociguat in 445 CTD-PAH patients (253 patients in active drug arm), the mean difference was -124 pg/mL (95% CI − 545 to − 297), favoring the control group, but the difference was not significant (Z = 0.58, p = 0.056, I^2^ = 55%, p = 0.14) (Fig. 3) [33, 44]. Among these 2 trials, Gaine et al. reported a median placebo-corrected change in serum NT-proBNP level of − 140 pg/mL (− 265, − 51), favoring the intervention arm [30]. However, the other RCT conducted with riociguat reported a mean increase of 274 ± 2576 pg/mL in the intervention group and 54 ± 778 pg/mL in the placebo group. In the third RCT with no data available for meta-analysis (AMBITION), the reported difference in the geometric mean ratio between the combination therapy group and the pooled monotherapy group was -30.4% (95% CI − 49.0 to − 5.2).

##### Hemodynamic parameters

*PVR*: Three RCTs reported the mean change in PVR after 12 weeks [28, 40, 44]. The treatment modalities used in these RCTs were sildenafil, epoprostenol iv, and riociguat. However, in the study comparing sildenafil and placebo in three different dose regimens, a significant difference was reported only in the sildenafil 20 mg subgroup but not in the 40 mg and 80 mg subgroups [40]. The meta-analyses of the trials revealed moderate heterogeneity between these trials (I^2^ = 71%, p < 0.002). The mean difference between groups was − 2.5 WU (95% CI − 3.67 to − 1.33, Z = 4.2, p < 0.001), favoring the intervention group (Fig. 3). The trial conducted with iv epoprostenol, which included patients with a higher baseline mean PVR compared to the other two studies, was the cause of heterogeneity. When this trial was excluded, the heterogeneity was significantly lower (I^2^ = 0%), and the mean difference decreased to − 1.59 WU (95% CI − 2.27 to − 0.91, Z = 4.57, p < 0.001).

*RAP*: Four RCTs conducted with sildenafil (20, 40, or 80 mg tid), epoprostenol iv, treprostinil sc, and riociguat (max 1.5 or 2.5 mg) reported mean change in RAP after 12 weeks as an outcome [28, 39, 40, 44]. There was a low heterogeneity between the trials (I^2^ = 0%, p = 0.55). Meta-analysis revealed a mean difference of − 1.24 mmHg (95% CI − 2.14 to − 0.33, Z = 2.68, p = 0.007), favoring the intervention group (Fig. 3). The meta-analysis of the trials conducted with iv epoprostenol and treprostinil revealed a higher mean difference (− 2.66, 95% CI − 4.33 to − 0.98, p = 0.002; I^2^ = 0%, p = 0.76).

*Cardiac index (CI)*: Three RCTs conducted with iv epoprostenol, sc treprostinil, and riociguat (max 1.5 or 2.5 mg) reported a mean change in CI at week 12 as an outcome [28, 39, 44]. In two of these trials (epoprostenol and treprostinil), the mean change in CI was significantly different, favoring treatment groups [28, 39]. In the other study (riociguat), the increase in mean change was higher in the treatment group, but they did not perform a test for significance (0.5 ± 0.5 vs. − 0.2 ± 0.6). In the meta-analysis, there was moderate heterogeneity between the trials (I^2^ = 43%, p = 0.16). A mean difference of 0.57 L/min/m^2^ (95% CI 0.39–0.75) was detected between the intervention and control groups, favoring the intervention group (Fig. 3).

#### Evaluation of quality bias

**Fig. 4:**
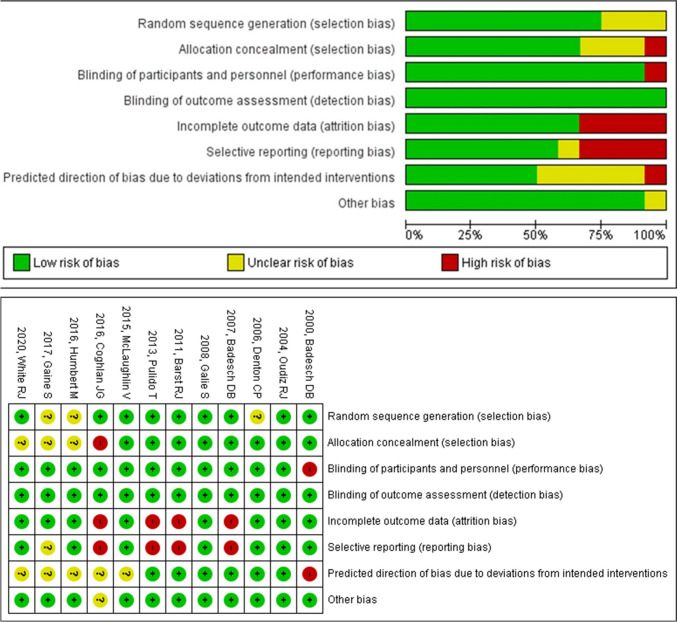
Risk of bias graph and summary: review of authors' judgments about each risk of bias item for each included study

The RoBs of the studies were generally low and are summarized in Fig. 4.

## Discussion

This meta-analysis, including 1837 patients, revealed that PAH-specific therapies are effective for most clinical (FC, 6-MWD, CW) and hemodynamic (PVR, RAP, and CI) outcomes in patients with CTD-PAH. However, the meta-analysis did not reveal a significant difference in the outcomes of NT-proBNP and survival. It should be noted that there was a limited number of studies providing data on NT-proBNP and survival in CTD-PAH patients (n = 2 of each). The short follow-up time may be a limitation for the assessment of survival (24 and 26 weeks per each).

A previous meta-analysis by Kuwana et al. had included nine studies, including single-arm studies, but did not include post hoc analyses of the RCTs and studies conducted after 2012 [23]. Another meta-analysis by Rhee et al. included 11 studies published between 2002 and 2013 [19]. This was a meta-analysis of the individual data of 827 patients with CTD-PAH acquired from the FDA. A more recent meta-analysis by Khanna et al., including 1267 patients with CTD, excluded studies with study groups of less than 30 patients and included 8 studies that reported long-term survival data of patients with CTD-PAH [20]. These differences in the studies included in the previous meta-analyses and our meta-analysis resulted in important differences in the pooled effect sizes of PAH-specific treatment modalities for CTD-PAH patients.

Improvement in WHO-FC and current FC status have prognostic importance in PAH; WHO-FC status is a domain of the PAH mortality risk assessment tool [1]. The proportion of patients with an improved WHO-FC was higher in the intervention groups compared to control groups (28% vs. 6%). An intervention group had a four times higher probability of having an improved WHO-FC compared to patients in the placebo groups. We could find only one study reporting comparative data in FC between IPAH and CTD-PAH, which was conducted with Tadalafil [43]. In this study, patients with idiopathic/heritable PAH had lower worsening rates of FC than patients with CTD-PAH. However, the FC improvement rates were similar.

Exercise capacity, assessed with 6-MWD, has a prognostic value in PAH [1, 46]. However, absolute or relative increases in 6-MWD do not correlate with mortality risk [47]. In addition, the minimal clinically important difference (MCID) of 6-MWD in CTD-PAH is unknown. The reported but unvalidated MCIDs ranged between 24 and 47 m in different studies [38, 48]. Our pooled analyses of the 12 subgroups for 6-MWD revealed a 36.2-m difference, which was above the MCID and favored PAH treatment, like the previous meta-analysis by Kuwana et al. (34.2 m). However, Rhee et al. and Khanna et al. reported a lesser increase that did not reach MCID (23.1 m and 20.4 m). The reported increase in 6MWD after PAH treatment was higher in IPAH patients compared to CTD-PAH patients in several studies [19, 23, 30, 40, 49]. However, the increase in 6-MWD reported by Khanna et al. in overall PAH patients was similar to the increase in CTD-PAH patients in this meta-analysis. The observed difference in treatment response between CTD-PAH and non-CTD-PAH patients underlines the importance of ensuring enough power to analyze CTD-PAH patients and reporting the results of CTD-PAH patients separately when designing future RCTs with PAH-specific agents.

Our analysis showed a 39% reduction in the risk of CW with PAH treatment. The risk reduction was more distinct with the combination therapies (46%). Khanna et al. reported a 36% reduction in the risk of clinical morbidity/mortality events. Survival was a domain of combined CW outcome in all studies reporting CW. However, the individual survival data were reported only in 2 studies. Our meta-analysis did not find the added benefit of PAH treatment in short-term survival.

Lower NT-proBNP levels in patients with SSc were associated with longer event-free survival. The GRIPHON (selexipag) trial reported a greater decrease in NT-proBNP levels in the intervention group. In the AMBITION trial, combination treatment (ambrisentan + tadalafil) resulted in a greater reduction in NT-proBNP level. We did not find a significant difference in absolute change in NT-proBNP levels at the follow-up between intervention and placebo groups in the pooled analyses of the two RCTs using selexipag and riociguat [33, 44]. These results are comparable with the COMPERA registry, which showed that a relative decline in NT-proBNP levels of more than 35% was an independent predictor of improved survival, whereas the absolute change in NT-proBNP levels was not associated with improved survival [50].

While PVR is mandatory in diagnosing PAH, it does not correlate with a change in exercise capacity or improved outcomes in patients with PAH. However, PVR has a certain correlation with the other indicators (6MWD, WHO FC, and CI) of risk, and it is an endpoint in many studies exploring the efficacy of PAH drugs and strategies. In contrast, RAP and CI are not diagnostic parameters for PAH, but they are important risk indicators of mortality [1]. About one-third of the RCTs in this meta-analysis have included hemodynamic data. Our meta-analysis revealed a significant mean placebo or monotherapy-corrected difference between groups for all three outcomes (PVR, RAP, and CI), favoring active treatment groups. The treatment effects significantly differed between dosing subgroups in SUPER-1 (sildenafil) and PATENT-1 (riociguat) studies for PVR and CI. In the SUPER-1 study, which included patients with IPAH (%60), CTD-PAH (%30), and other causes (%10), the mean decrease in PVR was higher in 40 mg and 80 mg tid subgroups but lower in the 20 mg tid subgroup compared to patients with CTD-PAH [40, 51, 52]. In contrast, improvement of PVR with a lower sildenafil dose was reported in some studies and suggested that a lower dose may be more selective for pulmonary vasculature [52, 53]. PVR decrease was lower in the PATENT-1 study (riociguat vs. placebo), which consisted mostly of patients with IPAH (60%), compared to the PVR decrease detected in the patients with CTD-PAH in our meta-analysis (− 1.8 vs. − 2.1 WU) [44, 54]. The reasons for the different dose or etiology-dependent responses of the same drugs in PAH must be elucidated.

Our meta-analysis revealed that RAP significantly decreased with treatment. However, epoprostenol (iv) and treprostinil (sc) dominate this beneficial effect, and the treatment options targeting pathways other than the prostacyclin pathway still lack evidence for decreasing RAP in CTD-PAH.

CI improvements were similar in the entire group and CTD-PAH subgroup in the PATENT-1 study. A study that mainly included IPAH patients (18/21) reported a slightly higher CI improvement in combination treatment with bosentan and iloprost (0.55 L/min/m^2^) than we identified in the meta-analysis (0.46 L/min/m^2^) [55]. The SERAPH study that included mostly IPAH patients reported a lesser improvement (0.3 L/min/m^2^, 95% CI 0.1–0.4) with bosentan and sildenafil [56]. In the SUPER study (sildenafil vs placebo) in which 60% had IPAH, placebo corrected mean difference with sildenafil 20 mg, 40 mg, and 80 mg tid were 0.21, 0.23, and 0.39 L/min/m^2^ respectively [51]. These results suggest that, in contrast to changes in exercise capacity, changes in hemodynamic parameters after PAH treatment were similar between patients with IPAH and those with CTD-PAH.

Today, upfront combination therapy has almost become a standard of care in patients with PAH. However, there is still insufficient evidence for upfront combination treatment in CTD-PAH. Two RCTs (AMBITION and TRITON) questioned the upfront combination therapy in CTD-PAH. The post hoc analysis of the AMBITION study revealed that the upfront combination of ambrisentan and tadalafil provided a risk reduction of 57% compared to monotherapy. The TRITON study compared the change in PVR after 26 weeks between triple and dual upfront combination therapies (selexipag + macitentan + tadalafil vs. macitentan + tadalafil). It showed significant improvement in PVR in both groups without significant difference between groups. Among the 18 RCTs with CTD-PAH, 12 RCTs may be considered to represent a sequential combination trial design because, in 6 of these studies, more than half of the included patients, and in the remaining 6 RCTs all of the included patients were receiving background PAH treatment [28, 30–37, 41, 43, 44]. However, published data are limited in interpreting the effects of combination therapy on most outcomes.

There are a number of issues that prevent making definite conclusions about CTD-PAH. The first one is that patients with SSc were included most frequently (59%) among CTDs in RCTs. Data on other CTD groups were very limited. We could not make a comparative analysis between CTD subgroups due to the lack of available data for meta-analysis. Patients with SSc and other CTDs may have different treatment responses [44, 57]. Therefore, the results of our meta-analysis may not be valid for all CTD subgroups. The second one, PAH diagnostic criteria, were not standardized in studies. Different PVR cut-offs were used as entry criteria. Another issue is that the involvement of the myocardium and lungs may cause PH and may not be apparent initially. Since these studies were not planned specifically for CTD-PAH patients, lung or myocardial involvement was not evaluated in detail in most studies at baseline or during follow-up. The different duration of the trials (ranging from 12 to 192 weeks), the prolonged period between the publication of the first and last RCT, and the possible differences in the background CTD therapies were other limitations.

In conclusion, this meta-analysis of RCTs demonstrates that modern PAH-specific treatments provide important clinical and hemodynamic benefits for CTD-PAH. However, the differences in inclusion criteria, problems in study design, and lack of studies conducted specifically for patients with CTD-PAH impair a reliable estimation of the effect size. Further studies conducted in CTD-PAH patients using appropriate outcome measures are needed to develop optimal management strategies for these patients.

### Supplementary Information

Below is the link to the electronic supplementary material.

Supplementary file1 (DOCX 731 KB)
