# Supplementary material for: Treatment of pulmonary arterial hypertension in patients with connective tissue diseases: a systematic review and meta-analysis
Source: Intern Emerg Med. 2024 Feb 20;19(3):731–43. doi: 10.1007/s11739-024-03539-1 (PMC11039558; doi:10.1007/s11739-024-03539-1)
Supplement: Supplementary file 1 — Supplementary file1 (DOCX 731 KB) [file 11739_2024_3539_MOESM1_ESM.docx]

**Table S1. Study characteristics of patients with CTD-PAH in the studies included in the meta-analyses**

| **First Author, Year** | **Official**  **acronym** | **Intervention** | | **Number of**  **Patients** | | **Age** | | **Female (%)** | | **Primary Outcome** | **Trial duration (weeks or months)** | **Etiology, n (%)** | | |
| --- | --- | --- | --- | --- | --- | --- | --- | --- | --- | --- | --- | --- | --- | --- |
|  |  | **Trial** | **Control** | **Trial** | **Control** | **Control** | **Trial** | **Control** | **Trial** |  |  | **SSc** | **SLE** | **Others** |
| Badesch DB, 2000 | - | Epoprostenol iv | Placebo | 55 | 55 | 53±13.1 | 57.3±10.3 | 91 | 82 | Δ6-MWD (meters) | 12 wk | 47 (80) | 16 (20) | 0 |
| Oudiz RJ, 2004 | - | Treprostinil sc | Placebo | 41 | 49 | 54±2 | 48 ± 2 | 93 | 88 | Δ6-MWD (meters) | 12 wk | 19 (45)** | 16 (38) | 7 (17) |
| Denton CP, 2006 | BREATHE-1 | Bosentan | Placebo | 44 | 22 | 57.7±12.6 | 49.7±12.7 | 86 | 77 | Δ6-MWD (meters) | 16 wk | 52 (79) | 8 (12) | 6 (9) |
| Badesch DB, 2007 | SUPER-1 | Sildenafil 20, 40, or 80 mg tid | Placebo | 62 | 22 | 52 ± 15 (20mg group) 50 ± 15 (40mg group) 54 ± 14 (80mg group) | 56 ± 14 | 83 | | Δ6-MWD (meters) | 12 wk | NR | NR | NR |
| Galie S, 2008 | AIRES 1 - 2 | Ambrisentan | Placebo | 81 | 43 | 53±14 in 5mg group (Aries-1)  49±16 in 10 mg group (Aries-1)  52±15 in 2.5 mg group (Aries-2)  50±16 mg in 5 mg group (Aries-2) | 48±16 in Aries-1  51±14 in Aries-2 | 80 | 78 | Δ6-MWD (meters) | 12 wk | NR | NR | NR |
| Barst RJ, 2011 | PHIRST-1 | Tadalafil 20mg or 40mg | Placebo | 36 | 16 | 49.8 (±14.7) (20mg group)  50.0 (±12.9) (40mg group) | 51.7 (±16.1) | 79 | 75 | Δ6-MWD (meters) | 16 wk | NR | NR | NR |
| Pulido T, 2013 | SERAPHIN | Macitentan 3 mg or 10 mg | Placebo | 143 | 164 | 44.5±16.26 (3mg group)  45.5±14.99 (10mg group) | 46.7±17.03 | 77 | | TTCW | 36 mo | NR | NR | NR |
| McLaughlin V, 2015 | COMPASS-2 | Bosentan 62.5-125 mg bid | Placebo | 43 | 45 | 52.9±15.4 | 54.7±15.7 | 76 | | Time to the first morbidity/mortality | 16 wk^Ψ^ | NR | NR | NR |
| Coghlan JG, 2017 | AMBITION | Amb. 10 mg + Tad. 40 mg | Amb. 10 mg or Tad. 40mg | 103 | 84 | 58.4 (12.3) (combination group)  57.9 (13.1) (pooled monotherapy group) | NR | 86 | 90 | TTCW | 12 wk^Ψ^ | 118 (63) | 17 (9) | 52 (28) |
| Humbert M, 2017 | PATENT 1- 2 | Riociguat up to 1,5 or 2,5 mg tid | Placebo | 151 | 66 | 49±16 (1,5mg group)  51±17 (2,5mg group) | 51±17 | 87 | | 6MWD (LS mean difference) | 12 wk | NR | NR | NR |
| Gaine S, 2017 | GRIPHON | Selexipag 200-1600 μg bid*** | Placebo | 167 | 167 | 51.8±14.1 | 52.8±15.0 | 93 | 87 | TTCW | 26 wk^Ψ^ | 170 (51) | 82 (24.5) | 82 (24.5) |
| White RJ, 2020 |  | Treprostinil 0.125 mg tid | Placebo | 94 | 84 | 45.6±15.7 | 44.8±15.4 | 79 | | TTCW | 24 wk^Ψ^ | NR | NR | NR |
| The number of patients with any PAH, including idiopathic pulmonary hypertension and associated pulmonary hypertension, Ω: intervention group, ¥: control group, ^Ψ^ for secondary outcomes.  *** 200-400 μg: 16.6%, 600-1000 μg: 27%, 1200-1600 μg: 44.9%  Amb: Ambrisentan,6-MWD: Six-minute walk distance, CI: cardiac index, CTD: connective tissue disease, EQ-5D, EuroQol five dimensions questionnaire, FC: functional classification, LPH: living with pulmonary hypertension questionnaire, iv: intravenous, mg: milligram, mPAP: mean pulmonary artery pressure, MVO2: mean venous oxygen saturation, NR: Not reported, NT-proBNP: N-terminal prohormone of brain natriuretic peptide PVR: pulmonary vascular resistance, QoL: quality of life, RAP: right atrial pressure, Tad: Tadalafil WHO: world heart organization | | | | | | | | | | | | | | |

**Table S2. Study characteristics of the studies** **included patients with PAH-CTD^¥^**
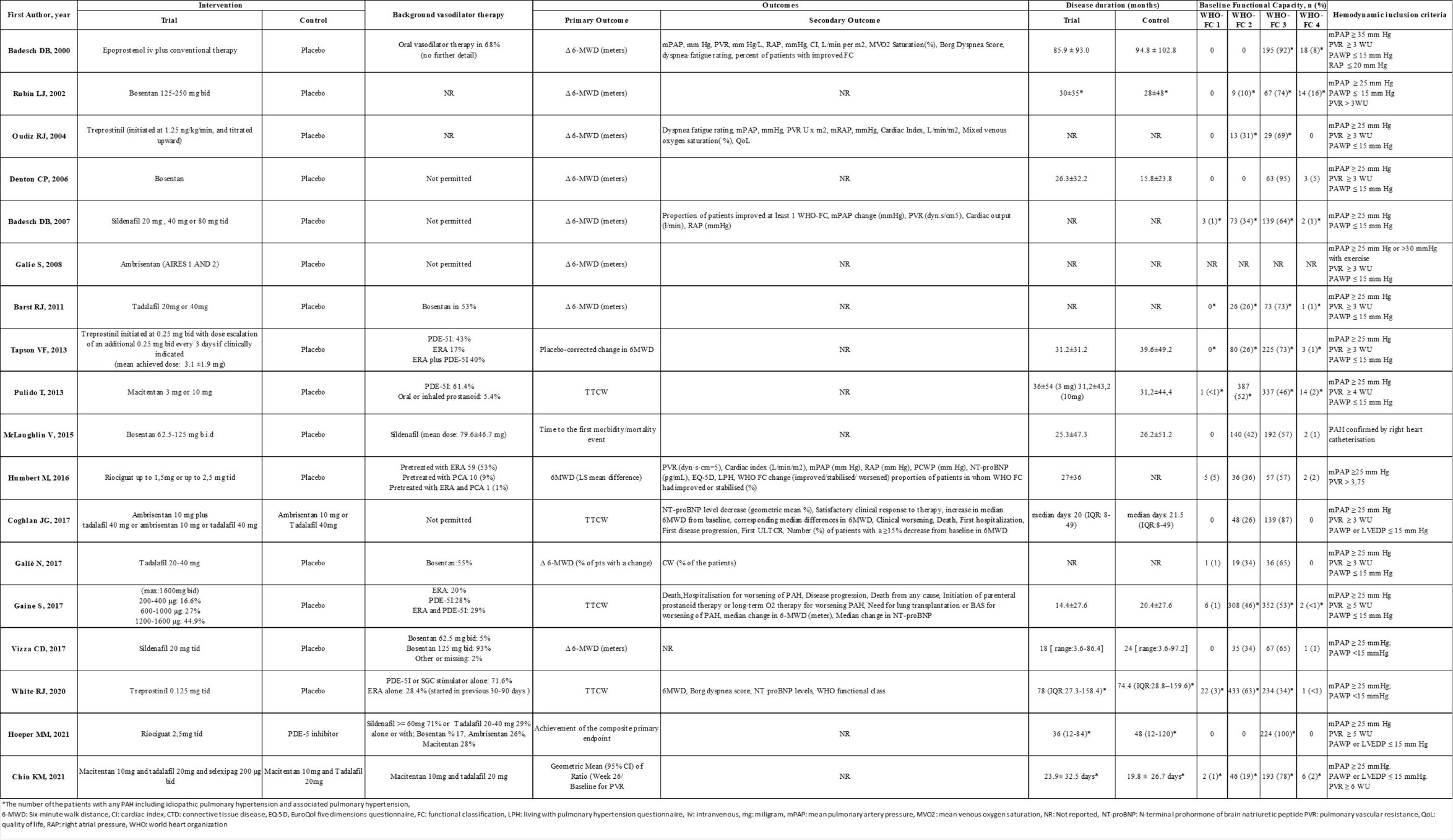


¥ The PAH studies reported outcomes for patients with CTD-PAH with or without available data for meta-analyses.

*The number of patients with any PAH, including idiopathic pulmonary hypertension and associated pulmonary hypertension,

6-MWD: Six-minute walk distance, CI: cardiac index, CTD: connective tissue disease, EQ-5D, EuroQol five dimensions questionnaire, FC: functional classification, LPH: living with pulmonary hypertension questionnaire, iv: intravenous, mg: milligram, mPAP: mean pulmonary artery pressure, MVO2: mean venous oxygen saturation, NR: Not reported, NT-proBNP: N-terminal prohormone of brain natriuretic peptide PVR: pulmonary vascular resistance, QoL: quality of life, RAP: right atrial pressure, WHO: world heart organization

**Table S3. Definitions of the clinical worsening used in the studies**

| **First author, year** | **Study Drug** | **Definition of clinical worsening** |
| --- | --- | --- |
| Rubin LJ, 2002 | BOSENTAN (BREATHE-1) | defined as the combined end point of death, lung transplantation, hospitalization for pulmonary hypertension, lack of clinical improvement or worsening leading to discontinuation, need for epoprostenol therapy, or atrial septostomy |
| Pulido T, 2013 | MACITENTAN (SERAPHIN) | Worsening of pulmonary arterial hypertension, initiation of treatment with intravenous or subcutaneous prostanoids, lung transplantation, or atrial septostomy or death from any cause up to the end of treatment. Worsening of pulmonary arterial hypertension was defined by the occurrence of all three of the following: a decrease in the 6-minute walk distance of at least 15% from baseline, confirmed by a second 6-minute walk test performed on a different day within 2 weeks; worsening of symptoms of pulmonary arterial hypertension; and the need for additional treatment for pulmonary arterial hypertension. Worsening of symptoms of pulmonary arterial hypertension included at least one of the following: a change from baseline to a higher WHO functional class (or no change in patients who were in WHO functional class IV at baseline) and the appearance or worsening of signs of right heart failure that did not respond to oral diuretic therapy. |
| McLaughlin V, 2015 | BOSENTAN ADDED TO SILDENAFIL (COMPASS-2) | Death from any cause, hospitalization for worsening PAH or start of intravenous prostanoid therapy, atrial septostomy, lung transplant, or worsening PAH.  Worsening PAH was defined as: 1) moderate or marked worsening of PAH symptoms on the PGSA together with the initiation of a subcutaneous or inhaled prostanoid or use of open-label bosentan; or 2) no change or mild worsening of PAH symptoms accompanied by a decrease in 6MWD by more than 20% from the previous visit or by more than 30% from the baseline visit, together with the initiation of a subcutaneous or inhaled prostanoid or use of open-label bosentan. Patients reporting improvement in the PGSA were not considered to have experienced a worsening PAH event. |
| Coghlan JG, 2016 | AMBRISENTAN / TADALAFIL  (AMBITION) | Death, hospitalization for worsening PAH (any hospitalization for worsening PAH, lung or heart/lung transplant, atrial septostomy or initiation of parenteral prostanoid therapy), disease progression (decrease of >15% from baseline 6MWD combined with WHO functional class III or IV symptoms at two consecutive visits separated by ≥14 days) or unsatisfactory long-term clinical response (any decrease from baseline 6MWD at two consecutive postbaseline clinic visits separated by ≥14 days and WHO functional class III symptoms assessed at two clinic visits separated by ≥6 months) |
| Gaine S, 2017 | Selexipag (GRIPHON) | Disease progression or worsening of PAH that resulted in hospitalization, initiation of parenteral prostanoid therapy or long-term oxygen therapy, the need for lung transplantation or balloon atrial septostomy, or death from any cause Disease progression was defined as a ⩾15% decrease in 6-min walk distance from baseline, confirmed by a second test on a different day, and worsening in World Health Organization (WHO) functional class (for patients in functional class II/III at baseline) or need for additional PAH therapy (for patients in functional class III/ IV at baseline) |
| White RJ, 2020 | TREPROSTINIL (extended-release tablets) vs Placebo | Clinical worsening (adjudicated) was defined as the occurrence of any 1 of the following events: Death (all causes) Hospitalization due to worsening PAH is defined as;  Non-elective hospitalization lasting at least 24 hours in duration caused by clinical conditions directly related to PAH and/or right heart failure or o Lung or heart/lung transplantation or o Atrial septostomy Initiation of an inhaled or infused PGI2 for the treatment of worsening PAH Disease progression (all criteria required): A decrease in 6MWD of at least 15% from baseline (or too ill to walk) directly related to PAH progression with other comorbidities ruled out, confirmed by two 6-Minute Walk Tests (6MWTs) performed on different days (confirmatory 6MWTs were required to be conducted within 30 days of the qualifying decrease in 6MWD; however, confirmatory 6MWTs that occurred outside of this window E8 were considered to have met the definition of clinical worsening and sent to the adjudication committee for review). Worsening of PAH symptoms, which included either: An increase in World Health Organization functional class (WHO FC) from baseline or Appearance or worsening of symptoms of right heart failure since baseline Unsatisfactory long-term clinical response (all the following criteria required): Randomized to receive study drug for at least 24 weeks o A decrease from baseline in 6MWD at Week 24 and beyond at 2 consecutive visits on different days (confirmatory 6MWTs were required to be conducted within 30 days of the qualifying decrease in 6MWD; however, confirmatory 6MWTs that occurred outside of this window were considered to have met the definition of clinical worsening and sent to the adjudication committee for review). Sustained WHO FC III or IV symptoms for at least 24 weeks consecutively |

**Figure S1. The meta-analysis of the clinical worsening of the combination therapies**


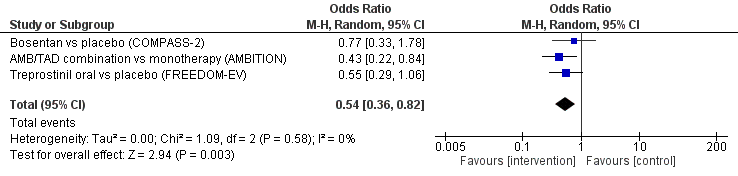


**The intervention and patient characteristics**

The pharmacological interventions were prostacyclin analogs in 4 studies (epoprostenol in 1 study and treprostinil in 3 studies), PDE-5 inhibitors in 4 studies (sildenafil and tadalafil in 2 studies each), ERA in 5 studies (bosentan in 3 studies, macitentan in 1 study, and ambrisentan in 1 study), riociguat in 2 studies, and selexipag in 2 studies. In one study, the combination of ambrisentan and tadalafil was compared with pooled monotherapy, and in another study, triple combination therapy was compared with dual therapy. The follow-up duration of selected trials ranged from 12 weeks to 192 weeks.

After the exclusion of the 6 studies without analyzable data for meta-analyses (1-6), the number of patients in the remaining 12 trials included in the meta-analyses was 1837 (7-18). The underlying CTD diagnosis was reported in 818 patients included in 7 studies. These diagnoses were SSc in 59% (n=480/818), SLE in 20% (n=164/818), and other CTDs in 21% of the patients (n=174/818) (1, 4, 7, 8, 12-14).

**Quality Assessment Details**

The RoB 2 considers random sequence generation, allocation concealment, blinding of participants and outcome assessors, deviations from the intended interventions, completeness of outcome data, selective reporting, and other biases for grading the quality of included studies.

1. Rubin LJ, Badesch DB, Barst RJ, Galie N, Black CM, Keogh A, et al. Bosentan therapy for pulmonary arterial hypertension. N Engl J Med. 2002;346(12):896-903.

2. Tapson VF, Jing ZC, Xu KF, Pan L, Feldman J, Kiely DG, et al. Oral treprostinil for the treatment of pulmonary arterial hypertension in patients receiving background endothelin receptor antagonist and phosphodiesterase type 5 inhibitor therapy (the FREEDOM-C2 study): a randomized controlled trial. Chest. 2013;144(3):952-8.

3. Galie N, Denton CP, Dardi F, Manes A, Mazzanti G, Li B, et al. Tadalafil in idiopathic or heritable pulmonary arterial hypertension (PAH) compared to PAH associated with connective tissue disease. Int J Cardiol. 2017;235:67-72.

4. Vizza CD, Jansa P, Teal S, Dombi T, Zhou D. Sildenafil dosed concomitantly with bosentan for adult pulmonary arterial hypertension in a randomized controlled trial. BMC Cardiovasc Disord. 2017;17(1):239.

5. Chin KM, Sitbon O, Doelberg M, Feldman J, Gibbs JSR, Grunig E, et al. Three- Versus Two-Drug Therapy for Patients With Newly Diagnosed Pulmonary Arterial Hypertension. J Am Coll Cardiol. 2021;78(14):1393-403.

6. Hoeper MM, Al-Hiti H, Benza RL, Chang SA, Corris PA, Gibbs JSR, et al. Switching to riociguat versus maintenance therapy with phosphodiesterase-5 inhibitors in patients with pulmonary arterial hypertension (REPLACE): a multicentre, open-label, randomised controlled trial. The Lancet Respiratory medicine. 2021;9(6):573-84.

7. Badesch DB, Tapson VF, McGoon MD, Brundage BH, Rubin LJ, Wigley FM, et al. Continuous intravenous epoprostenol for pulmonary hypertension due to the scleroderma spectrum of disease. A randomized, controlled trial. Annals of internal medicine. 2000;132(6):425-34.

8. Oudiz RJ, Schilz RJ, Barst RJ, Galie N, Rich S, Rubin LJ, et al. Treprostinil, a prostacyclin analogue, in pulmonary arterial hypertension associated with connective tissue disease. Chest. 2004;126(2):420-7.

9. Badesch DB, Hill NS, Burgess G, Rubin LJ, Barst RJ, Galie N, et al. Sildenafil for pulmonary arterial hypertension associated with connective tissue disease. The Journal of rheumatology. 2007;34(12):2417-22.

10. Barst RJ, Oudiz RJ, Beardsworth A, Brundage BH, Simonneau G, Ghofrani HA, et al. Tadalafil monotherapy and as add-on to background bosentan in patients with pulmonary arterial hypertension. The Journal of heart and lung transplantation : the official publication of the International Society for Heart Transplantation. 2011;30(6):632-43.

11. Pulido T, Adzerikho I, Channick RN, Delcroix M, Galiè N, Ghofrani HA, Jansa P, Jing ZC, Le Brun FO, Mehta S, Mittelholzer CM, Perchenet L, Sastry BK, Sitbon O, Souza R, Torbicki A, Zeng X, Rubin LJ, Simonneau G; SERAPHIN Investigators. Macitentan and morbidity and mortality in pulmonary arterial hypertension. N Engl J Med. 2013 Aug 29;369(9):809-18. doi: 10.1056/NEJMoa1213917. PMID: 23984728.

12. Coghlan JG, Galie N, Barbera JA, Frost AE, Ghofrani HA, Hoeper MM, et al. Initial combination therapy with ambrisentan and tadalafil in connective tissue disease-associated pulmonary arterial hypertension (CTD-PAH): subgroup analysis from the AMBITION trial. Annals of the rheumatic diseases. 2017;76(7):1219-27.

13. Denton CP, Humbert M, Rubin L, Black CM. Bosentan treatment for pulmonary arterial hypertension related to connective tissue disease: a subgroup analysis of the pivotal clinical trials and their open-label extensions. Annals of the rheumatic diseases. 2006;65(10):1336-40.

14. Gaine S, Chin K, Coghlan G, Channick R, Di Scala L, Galie N, et al. Selexipag for the treatment of connective tissue disease-associated pulmonary arterial hypertension. The European respiratory journal. 2017;50(2).

15. Galie N, Olschewski H, Oudiz RJ, Torres F, Frost A, Ghofrani HA, et al. Ambrisentan for the treatment of pulmonary arterial hypertension: results of the ambrisentan in pulmonary arterial hypertension, randomized, double-blind, placebo-controlled, multicenter, efficacy (ARIES) study 1 and 2. Circulation. 2008;117(23):3010-9.

16. Humbert M, Coghlan JG, Ghofrani HA, Grimminger F, He JG, Riemekasten G, et al. Riociguat for the treatment of pulmonary arterial hypertension associated with connective tissue disease: results from PATENT-1 and PATENT-2. Annals of the rheumatic diseases. 2017;76(2):422-6.

17. McLaughlin V, Channick RN, Ghofrani HA, Lemarie JC, Naeije R, Packer M, et al. Bosentan added to sildenafil therapy in patients with pulmonary arterial hypertension. The European respiratory journal. 2015;46(2):405-13.

18. White RJ, Jerjes-Sanchez C, Bohns Meyer GM, Pulido T, Sepulveda P, Wang KY, et al. Combination Therapy with Oral Treprostinil for Pulmonary Arterial Hypertension. A Double-Blind Placebo-controlled Clinical Trial. American journal of respiratory and critical care medicine. 2020;201(6):707-17.
